# Can timed up and go subtasks predict functional decline in older adults with cognitive impairment?

**DOI:** 10.1590/1980-5764-DN-2021-0111

**Published:** 2022-12-05

**Authors:** Maiary Martins Souza, Juliana Hotta Ansai, Danielle Chagas Pereira da Silva, Paulo Giusti Rossi, Anielle Cristhine de Medeiros Takahashi, Larissa Pires de Andrade

**Affiliations:** 1 Universidade Federal de São Carlos, Departamento de Fisioterapia, São Carlos SP, Brazil.; 2 Universidade Federal de São Carlos, Departamento de Gerontologia, São Carlos SP, Brazil.; 3 Universidade Federal de São Carlos, Programa de Pós-Graduação em Fisioterapia, São Carlos SP, Brazil.

**Keywords:** Alzheimer Disease, Mobility Limitation, Aged, Cognitive Dysfunction, Functional Status, Doença de Alzheimer, Limitação da Mobilidade, Idoso, Disfunção Cognitiva, Estado Funcional

## Abstract

Even in the early stages of cognitive impairment, older people can present important motor alterations. However, there are no studies that have investigated Timed Up and Go (TUG) and its subtasks in predicting impairment of functional capacity over time in this population.

**Objectives:**

The aim of this study was to verify if the TUG test and its subtasks can predict functional decline over 32 months in older adults with mild cognitive impairment (MCI) and mild Alzheimer's disease (AD).

**Methods:**

This is a prospective 32-month follow-up study, including at baseline 78 older adults (MCI: n=40; AD: n=38). The TUG and its subtasks (e.g., sit-to-stand, walking forward, turn, walking back, and turn-to-sit) were performed at baseline using the Qualisys Motion system. Functional capacity was assessed at baseline and after 32 months.

**Results:**

After follow-up, the sample had 45 older adults (MCI: n=25; AD: n=20). Of these, 28 declined functional capacity (MCI: n=13; AD: n=15). No TUG variable significantly predicted (p>0.05) functional decline in both groups, by univariate logistic regression analysis with the covariate gender.

**Conclusions:**

Although older adults with MCI and mild AD declined functional capacity, the TUG test and its subtasks could not predict this decline over 32 months.

## RESUMO

Mesmo nos estágios iniciais do comprometimento cognitivo, os idosos podem apresentar alterações motoras importantes. No entanto, não há estudos que tenham investigado o *timed up and go* (TUG) e suas subtarefas como preditores do comprometimento da capacidade funcional ao longo do tempo nessa população.

**Objetivos:**

O objetivo deste estudo foi verificar se o teste *timed up and go* (TUG) e suas subtarefas podem predizer o declínio funcional ao longo de 32 meses em idosos com comprometimento cognitivo leve (CCL) e doença de Alzheimer leve (DA).

**Métodos:**

Este é um estudo prospectivo de acompanhamento de 32 meses, que incluiu no início do estudo 78 idosos (CCL: n=40; DA: n=38). O TUG e suas subtarefas (sentar para levantar, caminhar para frente, virar, caminhar para trás e girar para sentar) foram realizados na linha de base pelo sistema Qualisys Motion. A capacidade funcional foi avaliada no início e após 32 meses.

**Resultados:**

Depois do seguimento, a amostra foi composta de 45 idosos (CCL: n=25; DA: n=20). Destes, 28 tiveram a capacidade funcional diminuída (CCL: n=13; DA: n=15). Nenhuma variável do TUG previu declínio funcional significativamente estatístico (p>0,05) em nenhum dos grupos, por meio da análise de regressão logística univariada com a covariável sexo.

**Conclusões:**

Embora os idosos com CCL e DA leve tenham tido sua capacidade funcional diminuída, o teste TUG e suas subtarefas não puderam prever esse declínio em 32 meses.

## INTRODUCTION

Alzheimer's disease (AD) is the most common type of dementia among older adults, causing impairment in cognitive abilities, which interferes with the functional capacity of the individual^1,2^. Another common clinical condition in aging is mild cognitive impairment (MCI), also known as minor neurocognitive disorder^2^. The MCI is a transitional phase between natural aging and dementia. About 20% of older adults are diagnosed with MCI in developing countries, with an annual progression rate to dementia between 30% and 40%^3^. Identification, assessment, and early intervention in these older adults with impairments in functional capacity are essential^2^.

One of the ways to predict functional decline in older adults is the Timed Up and Go (TUG) test^4,5^. The TUG subtasks characterize a set of actions performed in one's routine, fundamental for independent mobility^6^. Although the TUG is widely used in clinical practice, there is a lack of studies to verify if the test can predict functional capacity decline in older adults with MCI and mild phases of AD. Execution times greater than 12.47 s present a greater risk of falls in the elderly^7^.

Most studies use TUG to analyze the variable time and a few other kinematic and kinetic variables that are able to assess balance. In addition, the analysis of partial times in their different subtasks allows greater accuracy and sensitivity to small changes in functional capacity^8^. Mirelman et al. found that older adults with MCI present a TUG performance with greater irregularity of gait step, lower trunk movement during transition subtasks, and lower axial rotation in the turn subtask compared to cognitively preserved individuals^9^. However, no studies were found that associated functional capacity and performance of TUG subtasks in older adults with MCI and AD, especially in the mild phase. This information could be useful for improving knowledge about cognitive impairment, functional capacity, prevention measures, and screening for declining functional capacity in older adults with cognitive impairment.

This study is justified by the fact that, although some studies show that even in the early stages of cognitive impairment, older people already present important motor alterations^10^, so far there are no studies that have investigated TUG and its subtasks in predicting impairment of functional capacity over time in a population with cognitive impairment. This information would be important, since the TUG subtasks can be performed and reproduced even in older people with difficulty in understanding, such as the older people with MCI and AD in the light phase^11^. Thus, the objective of the present study was to verify if the analysis of the TUG test and its subtasks is capable of predicting the decline of functional capacity over 32 months in older adults with MCI and mild AD.

## METHODS

### Study design and participants

From a longitudinal analytical study, the functional capacity of mildly aged older adults with MCI and mild AD was investigated at two assessment times (M1=initial; M2=after 32 months). The project was approved by the Federal University of São Carlos (UFSCar) Research Ethics Committee (CAAE: 72774317.7.0000.5504). The study was carried out at the Research Laboratory of Older Adults Health (LaPeSI), UFSCar (São Carlos, São Paulo state, Brazil). Survey participants and caregivers who needed follow-up consultations were given detailed information about the study, including all procedures that would be performed. After clarifying the doubts, the signing of the Free and Informed Consent Form was requested.

The recruitment process took place between January and September 2015 and was widely disseminated throughout the city. To calculate the sample size, the rule of at least 10 cases of the outcome (success or failure, depending on which was rarer) for each independent variable used in the linear regression model was used^12^. Elderly people with cognitive complaints and diagnoses of AD were invited to participate in an initial assessment. The eligibility criteria of the sample were individuals aged 60 years and over, not institutionalized, and with the possibility of telephone contact.

After recruitment, the eligible volunteers participated in an evaluation to confirm the diagnosis of MCI or mild AD, in partnership with a neurologist and professor. Inclusion criteria were individuals who were able to walk alone for at least 10 m without aid devices, who were willing to participate in the proposed assessments, and who were admitted to one of the groups. Exclusion criteria were the presence of stroke with motor sequelae, neurological disorders that interfered in cognition other than MCI and AD, or mobility (Parkinson's disease, multiple sclerosis, amyotrophic lateral sclerosis), severe and uncorrected audiovisual disorders that made communication difficult during the tests, and older adults with moderate or advanced AD at the initial moment.

For the diagnosis of MCI in the evaluation or confirmation of this diagnosis prior to the study, the following criteria were used:

- cognitive complaint corroborated by the person or by an informant (a person who stayed with the older person for at least half the day, four times a week);
- objective cognitive decline, scoring a score of 0.5 by the Clinical Assessment of Dementia (CDR)^13^;
- normal general cognitive function for the level of education, assessed by the Mini-Mental State Examination (MMSE)^14^;
- preserved functionality, assessed by the Pfeffer Scale^15^; and
- unaltered cognition or functionality to meet dementia criteria^15^.

AD diagnosis prior to the study was confirmed according to the Diagnostic and Statistical Manual of Mental Disorders (DSM-IV TR)^16^. Through the CDR, only those with a score of 1, indicating the mild stage^12^, were included in the group.

### Measures

The first evaluation took place between January and September 2015. We use the following instruments:

- Anamnesis, composed of a questionnaire with sociodemographic and clinical characteristics, such as age, gender, falls in the last year^17^, years of study, use of drugs, body mass index (kg/m^2^), and presence of diseases. Volunteers could also count on the informant's help to answer these questions;
- Geriatric Depression Scale (cutoff score of 5 points to screen risk of depressive symptoms)^18^; and
- Minnesota Leisure-Time Physical Activity Questionnaire (assessment of the level of energy expenditure)^19^.

**Figure 1:**
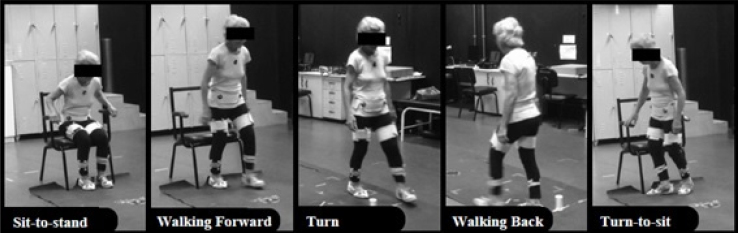
Performance on the subtasks of the timed up and go test in the Qualysis Pro Reflex system.

Volunteers were instructed to come in comfortable clothes and their usual closed shoes. Mobility was assessed by the TUG through the Qualysis Pro Reflex Motion Analysis System, consisting of seven 1280×1024 (1.3 megapixel) resolution cameras, and with an adapted chair^10^. The test involved, after the “go” command, getting up from the chair, walking 3 m, bounded by a cone at their usual speed, going back to the chair, and sitting down. The volunteers were instructed to start and end the test with the trunk leaning on the chair. Paused and standardized instructions were given along the test^10^. The TUG was subdivided into five subtasks: sit-to-stand, walking forward, turn, walking back, and turn-to-sit^20–22^ (Figure 1). The detection of TUG subtasks was performed according to the procedures demonstrated by Ansai et al.^11^, being performed by a single evaluator (intra-evaluator reliability above 0.72 in total). Data were captured by the Qualisys Track Manager acquisition software and transferred to the Visual-3D software for processing. The collection frequency was 120 Hz^22^. MATLAB software was used to detect, separate, and analyze TUG subtasks.

In TUG (total performance), the time spent using a stopwatch and the number of steps were analyzed. A step was considered when the heel was removed from the ground until it touched the ground again^23^. Regarding performances on TUG subtasks, time, trunk range of motion (pitch axis, i.e., flexion/extension), and average velocities of trunk (pitch axis) during the sit-to-stand subtask were analyzed. Data collected from walking forward and walking back subtasks were gait speed (GS), time, and length of the first step and number of steps. In the turn subtask, time, average velocity of trunk (yaw axis, i.e., rotation), and number of steps were collected. The same variables of the sit-to-stand and turn subtasks were analyzed in the turn-to-sit subtask^10,23^.

To assess functional capacity, the Pfeffer Scale^15^ of 10 items was used, showing a degree of independence for performing instrumental Activities of Daily Living (ADL). The minimum score is 0, and the maximum is 30 points. The higher the number of points, the greater the dependence of the older adult, considering the presence of impairment in functional capacity from a score of 5^15^.

The second assessment took place after 32 months, between September 2017 and May 2018. At this time, the functional capacity was assessed through the Pfeffer Scale and the Intercurrence Questionnaire was applied, in which the individual was asked about the occurrence of falls and other events during follow-ups, such as the number of hospitalizations, physical activity, physical therapy, and new diagnoses.

### Statistical analysis

Initially, a descriptive analysis of the data and a point and interval estimate of the parameters of interest were performed. For the analysis, a significance level of α=0.05 was adopted. Statistical tests were performed using the SPSS software (version 22.0). The Kolmogorov-Smirnov normality test was applied to all continuous variables to verify data distribution. Confirming the hypothesis of normality, the independent t-test was used to verify the difference between older adults with declining functional capacity (final Pfeffer − initial Pfeffer>0) and those with no decline in functional capacity (final Pfeffer − initial Pfeffer≤0) in both groups. The chi-square test was used to verify differences in sociodemographic characteristics. In addition, univariate logistic regression analysis was used to identify whether the TUG test, as well as its subtasks (variables available in Table 1), would be a good predictor of functional decline (final Pfeffer − initial Pfeffer>0). The confounding variable used was gender in univariate logistic regression models.

**Table 1:** Sociodemographic and clinical characteristics of sample at the baseline.

Characteristics	MCI Group			AD Group			MCI×AD
	No functional decline (n=12)	With functional decline (n=13)	p-value	No functional decline (n=5)	With functional decline (n=15)	p-value	p-value
Age, M±SD	72.3±4.3	76.1±8.3	0.169*	78.6±4.8	77.8±6.5	0.806*	0.067
Women, n (%)	11 (91.7)	11 (84.6)	0.588†	1 (20.0)	7 (46.7)	0.292†	0.001‡
Body mass index (kg/m^2^), M±SD	30.8±5.0	29.8±3.6	0.597*	26.8±4.7	27.2±5.5	0.886*	0.030‡
Years of schooling, M±SD	6.6±3.9	5.0±3.0	0.270*	5.8±2.4	5.7±5.3	0.969*	0.926
Total number of drugs, M±SD	7.3±4.6	8.0±6.9	0.757*	7.4±3.3	9.5±6.9	0.517*	0.415
History of falls at baseline, n (%)	7 (58.3)	7 (53.8)	0.581†	2 (40.0)	9 (60.0)	0.791†	0.634
Falls during 30 months, n (%)	5 (41.7)	10 (76.9)	0.108†	3 (60.0)	11 (73.3)	0.778†	0.402
GDS (0–15), M±SD	2.8±2.4	4.1±2.3	0.184*	3.0±1.8	2.6±2.5	0.755*	0.266
MMSE (0–30), M±SD	24.5±2.2	23.6±3.3	0.199*	19.0±8.7	17.4±4.7	0.434*	0.000‡
Minnesota (total score), M±SD	2281.4±2813.0	1188.4±953.3	0.241*	490.8±943.9	988.1±1266.8	0.878*	0.114
Pfeffer at baseline (absolute number) (0–30), M±SD	4.0±6.0	1.8±2.1	0.169*	13.2±11.6	12.3±10.5	0.806*	0.000‡

M±SD: mean±standard deviation; n (%): number of individuals (percentage); MCI: mild cognitive impairment; AD: Alzheimer's disease; kg/m^2^: kilogram/meter squared; GDS: Geriatric Depression Scale; MMSE: Mini-Mental State Examination; Minnesota: Minnesota Leisure Time Activities Questionnaire; *Analyzed by the independent t-test;

† Analyzed by chi-square test;

‡ p<0.05 (differences between subgroups for each group analyzed by the independent t-test or chi-square test).

## RESULTS

At baseline, we contacted 82 potentially eligible volunteers. Of these, four were excluded from the sample by presenting visual disturbance severe and uncorrected, AD in the moderate phase, motor sequel of stroke, and inability to ambulate alone. Thus, the initial sample consisted of 78 older adults, including 40 with MCI and 38 with mild AD.

**Figure 2:**
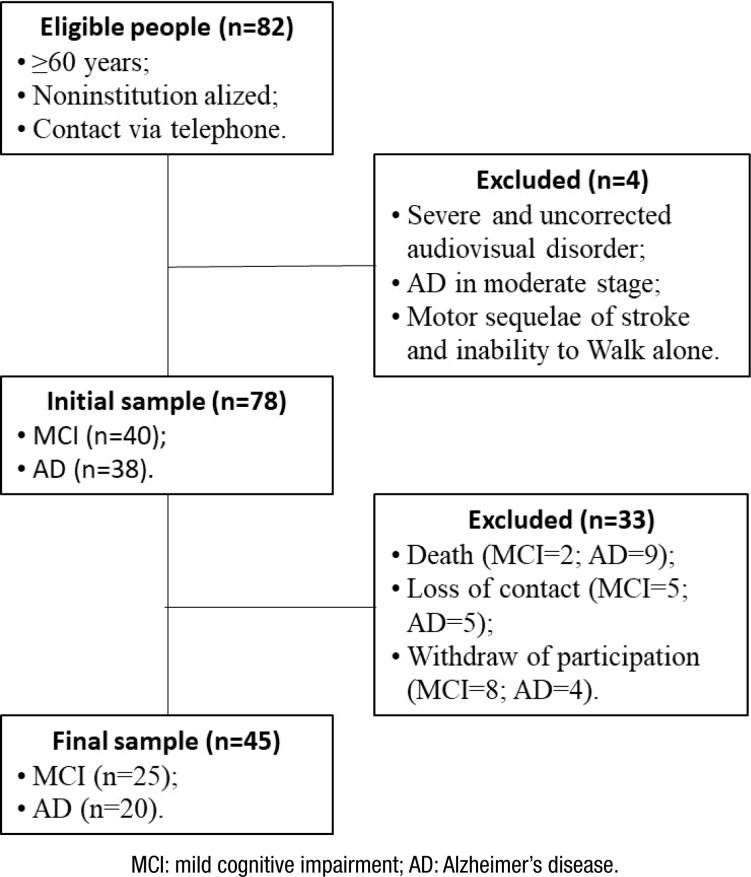
Flowchart of the sample.

In the second phase of the study, after 32 months, all 78 volunteers were again invited to participate. Of these, 11 people died, 10 were loss of contact, and 12 gave up participating in the survey, resulting in sample loss of 33 volunteers. Thus, the final sample consisted of 45 volunteers, i.e., 25 MCI and 20 AD (Figure 2).

Regardless of the group to which they belonged, volunteers were classified with and without functional decline, based on the analysis of functional capacity by Pfeffer^15^. In all, 28 volunteers scored higher than the initial assessment, characterizing a decline in functional capacity during the time segment studied (MCI=13; AD=15). Table 1 presents the sociodemographic and clinical characteristics of the sample, separated by groups and the presence of functional decline.

The groups with MCI, regardless of whether or not they had impaired functional capacity, were predominantly female volunteers (91.7% no functional decline and 84.6% with functional decline; MMSE no functional decline n=8 [M2=26.25±1.75], with functional decline n=9 [M2=22.44±5.57, p=0.085]). There was no statistically significant difference regarding sociodemographic variables and clinical characteristics between the MCI subgroups with and without functional decline, and the same was true for the AD group (Table 1).

Regarding the performance of the volunteers in TUG and its subtasks, it was observed that in the sit-to-stand subtask, both groups had similar averages regarding the duration of trunk acceleration movement. In the turn subtask, the volunteers with MCI performed the step-in terms of time, average speed, and number of steps with better performance compared to the AD group. In the walking back subtask, the values for the groups regarding walking speed and first step time were similar, and the length of the steps was equal. In the turn subtask, the MCI group also obtained values that demonstrate better performance compared to the AD group (Table 2).

**Table 2:** Timed up and go performance and its subtasks.

	Variable, M±SD	MCI group			AD group			MCI×AD
		No functional decline (n=12)	With functional decline (n=13)	p-value	No functional decline (n=5)	With functional decline (n=15)	p-value	p-value
Timed up and go performance	Total time (s)	14.2 (5.6)	12.7 (2.8)	0.419	15.9 (3.2)	14.0 (7.8)	0.618	0.372
	Number of steps	16.5 (2.9)	19.1 (6.3)	0.216	17.4 (4.1)	19.2 (5.8)	0.537	0.602
Sit-to-stand subtask	Time (s)	0.9 (0.4)	0.9 (0.2)	0.707	1.1 (0.3)	0.9 (0.4)	0.317	0.276
	Trunk range of motion, pitch axis (°)	20.5 (4.9)	24.7 (5.1)	0.057	18.6 (2.9)	20.8 (9.7)	0.624	0.198
	Trunk – average velocity, pitch axis (°/s)	44.2 (11.9)	47.7 (12.1)	0.478	29.7 (10.6)	35.2 (18.3)	0.533	0.004*
Walking forward subtask	First step – length (m)	0.2 (0.1)	0.2 (0.1)	0.602	0.2 (0.1)	0.3 (0.8)	0.439	0.134
	Number of steps	5.7 (2.5)	5.2 (1.2)	0.554	5.9 (1.0)	7.2 (5.9)	0.649	0.160
	First step – time (s)	0.6 (0.1)	0.6 (0.0)	0.690	0.7 (0.1)	0.6 (0.2)	0.554	0.593
	Gait speed (m/s)	0.4 (0.2)	0.5 (0.1)	0.843	0.3 (0.8)	0.3 (0.1)	0.281	0.001*
Turn subtask	Time (s)	2.3 (1.1)	2.0 (0.6)	0.433	2.1 (0.6)	3.2 (2.8)	0.396	0.085
	Trunk – average velocity, yaw axis (°/s)	73.2 (28.5)	74.9 (18.3)	0.854	62.8 (14.8)	43.5 (31.0)	0.204	0.001*
	Number of steps	4.5 (1.8)	4.2 (1.3)	0.594	4.2 (0.8)	4.7 (2.2)	0.656	0.452
Walking back subtask	First step – length (m)	0.3 (0.1)	0.3 (0.1)	0.700	0.2 (0.1)	0.1 (0.2)	0.458	0.211
	Number of steps	4.6 (2.5)	4.4 (1.3)	0.805	4.6 (0.9)	4.8 (1.8)	0.811	0.424
	First step – time (s)	0.7 (0.1)	0.6 (0.1)	0.730	0.6 (0.1)	1.8 (3.1)	0.419	0.116
	Gait speed (m/s)	0.6 (0.2)	0.6 (0.1)	0.783	0.5 (0.1)	0.9 (1.2)	0.468	0.495
Turn-to-sit subtask	Average velocity, pitch axis (°/s)	38.9 (9.7)	35.9 (10.7)	0.494	26.5 (5.4)	21.3 (20.2)	0.587	0.002*
	Average velocity, yaw axis (°/s)	42.4 (16.9)	43.2 (12.8)	0.902	36.3 (8.7)	28.3 (15.3)	0.287	0.008*
	Time (s)	2.4 (1.6)	2.0 (0.6)	0.431	2.5 (0.5)	2.7 (1.8)	0.796	0.087
	Trunk range of motion, pitch axis (°)	50.8 (5.6)	53.0 (7.8)	0.452	47.6 (7.5)	45.3 (17.8)	0.784	0.096
	Number of steps	3.9 (0.9)	3.7 (0.8)	0.763	4.5 (0.5)	3.9 (2.1)	0.561	0.451

M±SD: mean±standard deviation; MCI: mild cognitive impairment; AD: Alzheimer's disease; °: degree; s: seconds; m: meter; p>0.05 for all analyses by the independent t-test (both in the MCI group and the AD group).

In the logistic regression analysis, no mobility variable was significantly associated with functional decline, neither in the MCI nor in the AD groups (Table 3).

**Table 3:** Univariate mobility predictors of functional decline in participants with mild cognitive impairment and Alzheimer's disease, by the logistic regression analysis.

Measures		MCI group (n=25)		AD group (n=20)	
		OR (95%CI)	p-value	OR (95%CI)	p-value
Timed up and go performance – Total time (s)		0.914 (0.745–1.121)	0.389	0.982 (0.843–1.145)	0.820
Sit-to-stand subtask	Time (s)	0.564 (0.049–6.434	0.645	0.387 (0.026–5.757)	0.490
	Trunk range of motion, pitch axis (°)	1.198 (0.985–1.458)	0.071	1.029 (0.902–1.173)	0.674
	Trunk – average velocity, pitch axis (°/s)	1.032 (0.959–1.110)	0.402	1.014 (0.945–1.088)	0.702
Walking forward subtask	First step – length (m)	0.249 (0.001–108.118)	0.654	1.031 (0.723–1.470)	0.866
	Number of steps	0.860 (0.550–1.345)	0.509	1.148 (0.777–1.695)	0.488
	First step – time (s)	0.068 (0.000–194.213)	0.508	0.075 (0.000–75.308)	0.463
	Gait speed (m/s)	2.126 (0.026–171.993)	0.736	0.000 (0.000–83.329)	0.163
Turn subtask	Time (s)	0.641 (0.246–1.667)	0.361	1.594 (0.668–3.806)	0.294
	Trunk – average velocity, yaw axis (°/s)	1.005 (0.970–1.041)	0.768	0.943 (0.872–1.020)	0.144
	Number of steps	0.839 (0.492–1.431)	0.520	1.136 (0.652–1.981)	0.652
Walking back subtask	First step – length (m)	0.322 (0.001–115.496)	0.706	34.542 (0.002–701132,955)	0.484
	Number of steps	0.950 (0.629–1.436)	0.808	1.396 (0.635–3.069)	0.407
	First step – time (s)	0.315 (0.001–67.252)	0.673	4.677 (0.022–1006.629)	0.573
	Gait speed (m/s)	1.617 (0.027–95.097)	0.817	0.236 (0.003–20.101)	0.524
Turn-to-sit subtask	Average velocity, pitch axis (°/s)	0.961 (0.878–1.052)	0.390	0.936 (0.810–1.083)	0.374
	Average velocity, yaw axis (°/s)	1.011 (0.951–1.074)	0.729	0.928 (0.834–1.033)	0.173
	Time (s)	0.605 (0.262–1.401)	0.241	1.143 (0.599–2.182)	0.685
	Trunk range of motion, pitch axis (°)	1.039 (0.915–1.180)	0.554	0.986 (0.916–1.062)	0.708
	Number of steps	0.732 (0.258–2.072)	0.556	0.905 (0.448–1.827)	0.780

MCI: mild cognitive impairment; AD: Alzheimer's disease; °: degree; s: seconds; m: meter; p>0.05 for all analyses (both in the MCI Group and the AD Group); OR: odds ratio; CI: confidence interval, adjusted by gender.

## DISCUSSION

TUG and its subtasks did not allow greater precision in the evaluation of older adults with MCI or in mild phase of AD, rejecting the initial hypothesis of the present study that TUG could be a more sensitive test for small functional changes in this population. However, studies investigating whether alterations in physical tests, especially TUG, may predict functional alterations in older adults with cognitive impairment have not yet been found in the literature.

In a cohort study with older adults after anatomical lesions and requiring only minor outpatient procedures, it was observed that the use of TUG in older adults can help to identify individuals with bone frailty and at risk of functional decline^24^. Another study identified that the time to perform TUG was similar between older people with preserved cognition and MCI, but the quality to perform the test was different. This shows that there are motor-cognitive interactions already in individuals with MCI, i.e., at-risk stages for the development of dementia^25^. Zidan et al. verified that TUG is superior to the GS test in predicting multiple geriatric outcomes, including a decline of functional capacity, being able to predict the decline in health, the difficulty of performing ADL, and falls in community-dwelling older adults^26^.

In this study, although there was no significant difference in the relationship between TUG and functional capacity, it was possible to observe that the older adults who showed a decline in functional capacity over the 32 months had different performance in the sit-to-stand, walking back, and turn subtasks. In the sit-to-stand subtask, the mean achievement speed was higher in both groups that declined functional capacity. The sit-to-stand subtask is crucial for survival. Therefore, it is important to know that when it is compromised, it can interfere with the performance of ADL. This result may be partly explained by the association between cognitive impairment and lower limb function of the volunteers. The ability to get up from the chair involves complex factors where it is necessary to move the center of mass forward while still sitting, acceleration in the posteroanterior and vertical planes, push-off, and finally stabilization once the position standing is reached^8^.

In the walking back subtask, the step length was shorter when compared to the older adults who did not decline in terms of functional capacity, especially in the AD group. The aging process associated with the presence of neurodegenerative diseases, such as AD, aggravates gait automatism and increases balance deficit. In the mild phase of AD, the modulation of the locomotor pattern is increased, making gait more cautious^26,27^. Impairment in balance causes this caution to occur during walking, decreasing the length of the step and longer stay in double support, especially when they are preparing to perform a more complex activity such as, in the case of the present study, turn to sit down. These strategies adhered to by the older adults aim to reduce risks and maintain safety while walking^26,27^. Fear of falling can restrict individuals’ activities, leading them to a decline in functional capacity accompanied by decreased quality of life.

In the turn subtask, it was observed that the volunteers with mild AD who had declined in functional capacity took longer to perform it. With aging, gait demands more attention and resources, reflecting the need for different cognitive mechanisms for its proper control and performance^10^. Thus, it seems that the greater the cognitive impairment, the greater the demand to perform a given task, directly interfering with functional capacity. When observing the performance of TUG, the total number of steps was higher in individuals who reduced functional capacity, regardless of the group to which they belonged. All these findings are in agreement with the study by Mirelman et al. who found that individuals with cognitive impairment show greater irregularity of gait step, lower trunk movement during transition subtasks, and lower axial rotation during the turn subtask compared to cognitively preserved individuals^9^.

Subtle motor impairments are present in the transition from mild to moderate phases and worse performance in performing basic ADL in advanced AD^27^. Progress from explicit memory deficit to processing memory would explain the initial decline in the performance of instrumental ADL in people in the mild phase of DA. As the disease progresses, impairments in other cognitive abilities occur that further compromise basic activities^27^. The Pfeffer Scale used in this study evaluates items related to instrumental activities, while the TUG is a physical and mobility test. This fact may partly explain our results, where functional decline assessed by TUG was not sensitive to predict functional decline assessed by Pfeffer.

The final study sample consisted of 45 older adults, which was represented by the majority of females. According to Elahi and Miller, it is the gender that is most susceptible to the acquisition of dementia syndromes, such as MCI and AD^28^. In addition, other factors consistent with the literature were found, such as: a) prevalence of low level of education in the functionally declining group^29^, and b) higher hospitalization rate in the groups with functional decline, which may be correlated with the increase in the number of falls from M1 to M2 of all groups, except MCI, which did not decline functionally^30^. The number of volunteers who performed physical activities in the group that did not decline functionally was lower than the group that declined functionally, although they were advised to remain physically active^31^.

An important point to note is that the present study was followed up for 32 months, which was not the case with other studies found in the literature. Considering the follow-up time, a greater impairment of functional capacity was expected, especially in the group with the highest cognitive impairment.

This study is limited by the small sample size due to outcomes over time and the fact that we used the MMSE only as a cognitive assessment instrument. It also has a limitation in the fact that Pfeffer is a scale that applies to the caregiver in relation to the older adult; besides that, as previously mentioned, it evaluates the instrumental activities and not the functional capacity in general. However, it is important to note that the caregiver who responded to the instrument was the person who spent most of the time with the volunteer. In addition, Pfeffer is a scale widely used in clinical practice, and further investigations with these instruments may contribute to the knowledge of professionals who use it. It was also possible to observe a significant limitation of the absence of studies that discuss about the prediction of functional decline related to tests in older adults, mainly with MCI and AD. In contrast, as a strong point, it is important to emphasize that this is a longitudinal study and the first to address whether the TUG subtasks are related to functional decline.

Further longitudinal follow-up studies observing older adults with MCI and AD may provide clinical information on each TUG subtask, especially on the impact of these consequences on the individual's functional capacity.
